# Lymphangiogenesis and Lymphatic Remodeling Induced by Filarial Parasites: Implications for Pathogenesis

**DOI:** 10.1371/journal.ppat.1000688

**Published:** 2009-12-11

**Authors:** Sasisekhar Bennuru, Thomas B. Nutman

**Affiliations:** Laboratory of Parasitic Diseases, National Institute of Allergy and Infectious Diseases, National Institutes of Health, Bethesda, Maryland, United States of America; Case Western Reserve University, United States of America

## Abstract

Even in the absence of an adaptive immune system in murine models, lymphatic dilatation and dysfunction occur in filarial infections, although severe irreversible lymphedema and elephantiasis appears to require an intact adaptive immune response in human infections. To address how filarial parasites and their antigens influence the lymphatics directly, human lymphatic endothelial cells were exposed to filarial antigens, live parasites, or infected patient serum. Live filarial parasites or filarial antigens induced both significant LEC proliferation and differentiation into tube-like structures *in vitro*. Moreover, serum from patently infected (microfilaria positive) patients and those with longstanding chronic lymphatic obstruction induced significantly increased LEC proliferation compared to sera from uninfected individuals. Differentiation of LEC into tube-like networks was found to be associated with significantly increased levels of matrix metalloproteases and inhibition of their TIMP inhibitors (Tissue inhibitors of matrix metalloproteases). Comparison of global gene expression induced by live parasites in LEC to parasite-unexposed LEC demonstrated that filarial parasites altered the expression of those genes involved in cellular organization and development as well as those associated with junction adherence pathways that in turn decreased trans-endothelial transport as assessed by FITC-Dextran. The data suggest that filarial parasites directly induce lymphangiogenesis and lymphatic differentiation and provide insight into the mechanisms underlying the pathology seen in lymphatic filariasis.

## Author Summary

The nematode parasites *Brugia malayi* and *Wuchereria bancrofti* are the major organisms responsible for lymphatic filariasis. Lymphatic filariasis is characterized by the dysfunction of the lymphatics that can lead to severe (and often) irreversible lymphedema and elephantiasis. Current advances in distinguishing blood vascular from lymphatic endothelial cells have allowed the direct study of the interaction between live filarial parasites and their lymphatic niche. In the quest towards understanding parasite-lymphatic endothelium interactions, we observed that the filarial antigens have a specific but differential stimulatory capacity towards the lymphatics and cause them to differentiate into tube-like vascular networks *in vitro* that resemble the formation of collateral lymphatics *in vivo*. This was a lymphatic-specific phenomenon, as the filarial parasites or antigen did not exhibit similar effects on the human umbilical vein endothelial cells. The differentiation of the lymphatic endothelial monolayers into vascular networks was not dependent on typical markers of lymphangiogenesis but rather involves the matrix metalloproteases and their inhibitors that suggest lymphatic matrix remodeling rather than rendering of the lymphatics hyper-permeable as has been postulated previously.

## Introduction

Among the clinical manifestations of human infection with the filarial nematodes *Wuchereria bancrofti* and *Brugia malayi*, the most debilitating are those associated with lymphatic dysfunction and/or obstruction (e.g. lymphedema, elephantiasis). It is known that the establishment of the adult filarial parasite within the lymphatic vessels of the host triggers a cascade of events that leads to abnormalities in lymphatic integrity and function. In addition, the tissue tropism of these lymphatic-dwelling parasites suggest that the host provides cues to the parasite that provide developmental signals to the parasites [1]. Individuals infected with the lymph-dwelling filariae can develop tissue scarring and fibrosis within and around the lymphatic vessels resulting in permanent and characteristic pathology manifested clinically by irreversible lymphedema or elephantiasis [2],[3]. An extensive body of work supports the notion that some of the severe pathology is immune mediated [4],[5],[6],[7] but that lymphatic dilatation and alterations in lymphatic flow may be parasite-derived and unrelated to the adaptive immune response. Indeed, filaria-infected SCID mice have been shown to exhibit lymphatic dilatation [8],[9], a process that could be reversed by removing or killing the worms [10],[11]. Moreover, lymphoscintigraphy studies showed that patients with subclinical disease have considerable structural abnormalities and aberrant lymph flow [12],[13],[14], and ultrasonography has suggested that the anatomic location of the worm nests changes very little over time but that lymphangiectasia and dilatation are not restricted to the anatomic location of the adult worm nests [15],[16],[17]. In addition, in histologic studies of lymph nodes taken from patients with bancroftian filariasis, there were intact adult filarial worms with little or no associated inflammation [18],[19]. Together, these observations suggest a primary role of the parasites or parasite-encoded proteins in modulating lymphatic integrity and function. It has been speculated that, with chronic infection, it is the death of the adult worms (be it immune mediated or just intrinsic worm lifespan) and the release of parasite antigens (or Wolbachia) that induce proinflammatory responses that render a poorly functioning (and dilated) patent lymphatic completely obstructed [20].

Based on the available animal models, it has been assumed that the filarial parasites promote lymphangiogenesis, a developmental process that involves lymphatic endothelial proliferation followed by differentiation (tube formation). *In vitro* studies of parasite-endothelial interactions have been limited to those using human umbilical vein endothelial cells (HUVEC) [21] that differ quite extensively from lymphatic endothelial cells (LEC). In the present study, we have examined directly the influence filarial parasites have on LEC function and lymphangiogenesis. We demonstrate that filarial proteins do stimulate the LEC to undergo proliferation and differentiate into tube-like structures that involves matrix metalloproteases and tissue inhibitors of matrix metalloproteases. We further demonstrate that, contrary to data in murine models, the filarial parasites render lymphatics less (rather than hyper-) permeable and suggest possible new mechanisms underlying filarial-induced lymphedema.

## Results

### Filarial antigens induce differential proliferation of LEC

**Figure 1:**
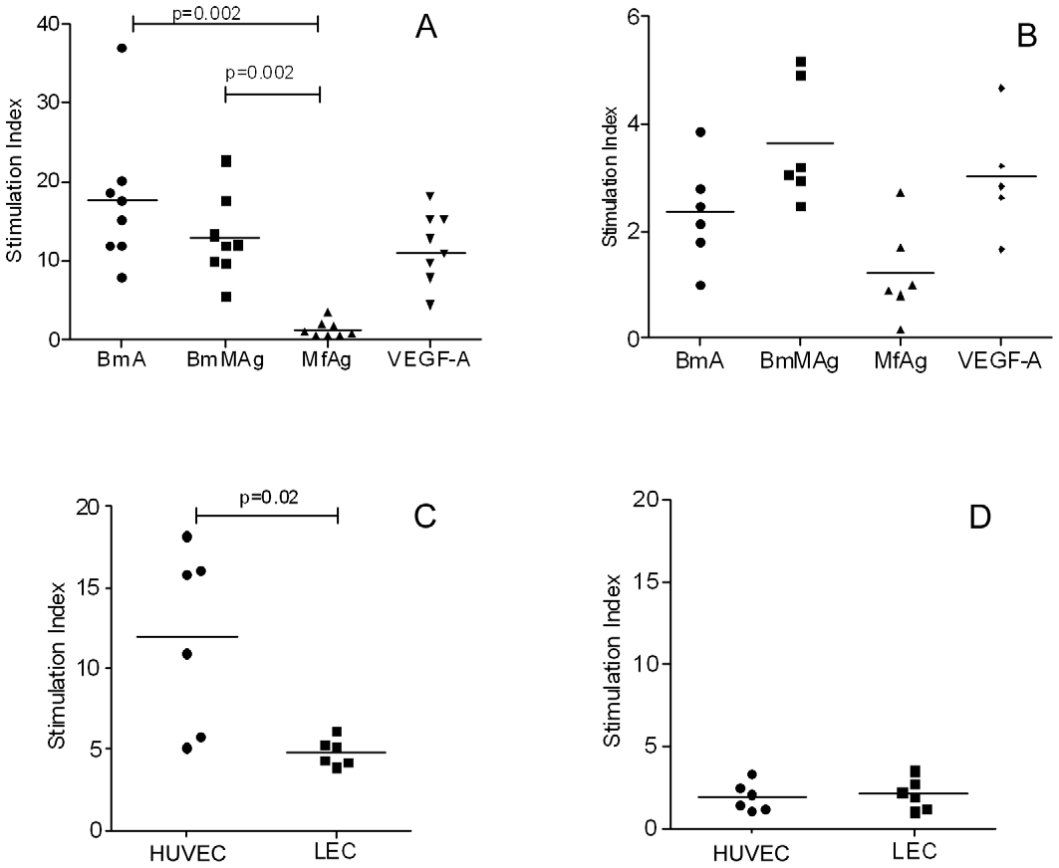
Filarial antigen-induced proliferation. LEC at 5×10^3^ were seeded in 96-well tissue culture plates in endothelial basal media and stimulated with optimized concentrations (10 µg/ml) of BmA, BmMAg, MfAg, and VEGF (25 ng/ml or 10ng/ml). Data are expressed as stimulation index over the unstimulated controls for each set of cultures and the horizontal bar is the geometric mean. Panels A and B show the response of LEC (Panel A) and HUVEC (Panel B) to the stimuli shown on the x-axis. Panels C and D show the proliferative effect on HUVEC and LEC of schistosome egg antigen (SEA Panel C) and of schistosome adult antigen (SWAP Panel D).

Analysis by flow cytometry and quantitative reverse transcriptase real-time PCR (qRT-PCR) demonstrated that the LEC were pure and possessed all of the characteristics of lymphatic endothelium (**Figure S1**). To evaluate the effect of filarial antigens on LEC proliferation, we stimulated LEC with stage-specific filarial antigens and measured proliferation at 96 h. As shown in **Figure 1A**, antigen derived from adult *Brugia malayi* parasites (BmA) or from adult male *B. malayi* parasites (BmMAg) induced proliferation of LEC with stimulation indices ranging from 8 to 35 and did so optimally at a concentration of 10 µg/ml (optimization data not shown). In contrast, antigens from microfilariae (MfAg) failed to induce proliferation at levels comparable to adult antigens. The extent of proliferation induced by BmA or BmMAg was comparable to that induced by recombinant soluble human vascular endothelial growth factor (VEGF)-A at 25 ng/ml used as a positive inducer of proliferation.

To address the specificity of this observed parasite-induced proliferation, both LEC and HUVEC, as a model representative of blood vascular endothelial cells were stimulated with the optimal concentrations of BmA, BmMAg, and MfAg for 96 h (based on preliminary dose and time-point assays) and the extent of proliferation quantified. As shown in **Figure 1B**, adult filarial antigens failed to induce proliferation of HUVEC, in marked contrast to the proliferation induced by these antigens in LEC. These data suggest that the filaria-induced EC proliferation was more specific to LEC compared to the HUVECs.

To address further the specificity of this response, non-filarial helminth antigens were tested. Because schistosome egg antigen (SEA) had been shown previously to induce proliferation of the HUVEC [22],[23], we examined the response of crude soluble extracts of *Schistosoma mansoni* adults (SWAP) and SEA on LEC and HUVEC proliferation. As seen, SEA induced proliferation of HUVEC but not LEC (**Figure 1C**), while SWAP failed to induce proliferation of either LEC or HUVEC (**Figure 1D**). Together, our data suggest that adult filarial antigens specifically induce LEC proliferation.

### Filarial serum promotes proliferation of LEC

**Figure 2:**
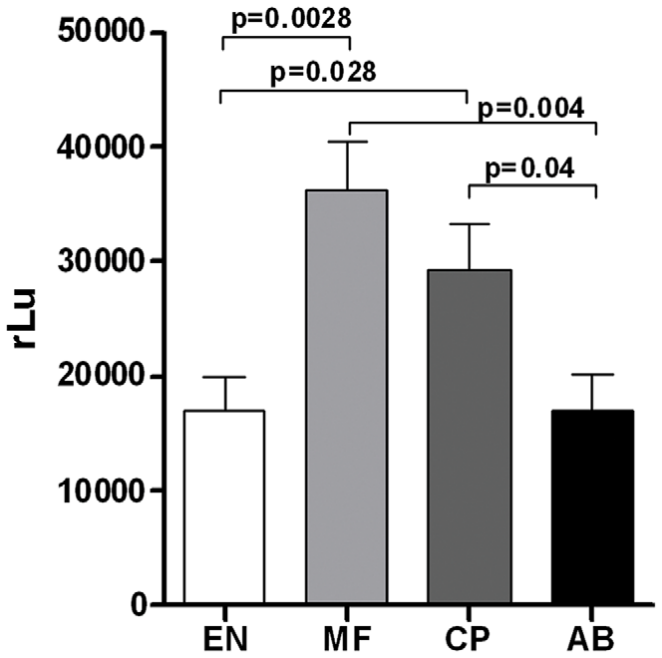
Filarial serum/plasma induced proliferation. LEC cultured with 1% pooled plasma from different patient groups (EN, MF, CP, AB) and proliferation measured by uptake of BrDU at 96 h. Data are expressed as relative luminescence units (rLu) over media controls and are expressed as the mean (+95% CI).

Having observed that filarial antigens induce LEC proliferation, we evaluated the potential of plasma (containing circulating filarial antigens and host angiogenic factors) to stimulate LEC proliferation. Using pooled plasma from filaria-uninfected endemic normal (EN) individuals, microfilaria-positive (MF) individuals with asymptomatic (or subclinical) infection, individuals with chronic lymphatic obstruction (CP), or AB serum from uninfected individuals (**Figure 2**), we were able to demonstrate that plasma from patients with lymphatic filariasis promoted LEC proliferation in contrast to that seen for uninfected EN plasma or control (AB) serum. As can be seen, the MF and CP plasma exhibited a significant increased proliferation compared with the EN plasma (*p* = 0.0028 and *p* = 0.028, respectively) or the AB serum (*p* = 0.0048 and *p* = 0.04, respectively). There was no difference in the proliferative capacity between the EN plasma or the AB serum. These data implicate filarial antigens and or soluble angiogenic factors circulating in the serum/plasma as having the capacity to induce LEC proliferation.

### Microfilarial antigens induce tube formation by LEC *in vitro*

**Figure 3:**
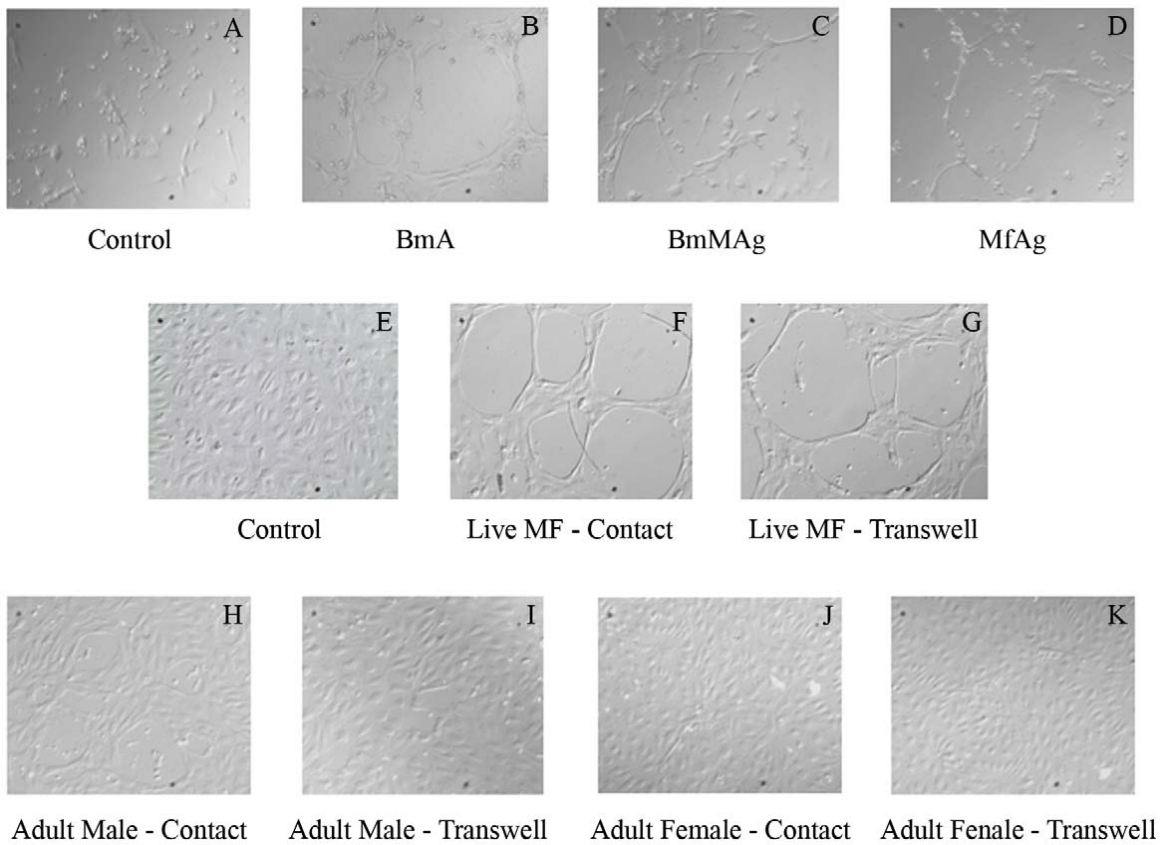
Microscopic images of filaria-induced lymphatic tube-like structure formation. LEC were seeded in endothelial basal media (Panels A and E) and stimulated with BmA (B), BmMAg (C), and MfAg (D), live microfilariae (F), live adult males (H), adult females (J) in contact or in transwells with live microfilariae (G), adult males (I), or adult females (K). (Magnification 20× (Panels A–D), 40× (Panels E–K)).

Because patent (microfilaraemic) filarial infection has been associated with lymphatic dilatation in both animal models and in human infection, we examined the effect of microfilarial antigen and live microfilariae (Mf) on LEC differentiation. When cultured in the presence of microfilarial antigens, the LEC were found to differentiate and form lymphatic tube-like structures when compared with unstimulated controls. Among the antigen preparations tested, MfAg was the most potent inducer of LEC differentiation, a process that could be observed as early as 48 h; in contrast, adult antigens could induce similar tube-like structures but did so only after 96 h (**Figure 3, A–D**). More importantly, live parasites (microfilariae, adult males, adult females) were also capable of inducing LEC differentiation. Live microfilariae (**Figure 3F**), compared with media controls (**Figure 3E**), induced LEC to form early tube like structures by 48–72 h in culture, whereas the adult male or female parasites induced tube formation only after 96 h in culture and did so in a limited and less complete fashion (**Figure 3H and 3J**). Increasing the number of live adult parasites did not appear to augment LEC differentiation (data not shown). To evaluate whether tube formation was contact dependent or independent, LEC were cultured with the parasites in transwell inserts. Interestingly, microfilariae co-cultured with LEC in transwell inserts also induced tube formation (**Figure 3G**), while the adult parasites in transwells did not (**Figure 3I and 3K**). These data suggest that this contact-independent LEC differentiation is primarily mediated by the excretory-secretory products of the parasites and of microfilariae most prominently.

### MfAg-induced tube formation is independent of expression of known lymphangiogenic molecules

**Figure 4:**
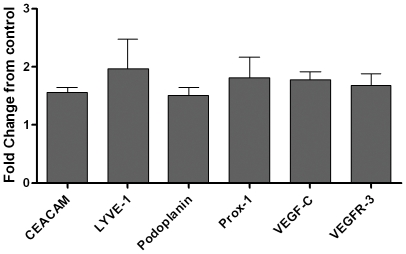
Expression of lymphangiogenic markers upon stimulation with MfAg by LEC as assessed by qRT-PCR. Data are expressed as the mean (+95% CI) fold change from unstimulated controls of 4 independent experiments.

Formation of lymphatic capillaries *in vivo* (in animal models) has been shown to be regulated by the expression of Prox-1 and podoplanin, among other molecules [24]. To evaluate whether the capillary tube formation induced by microfilariae involved upregulation of these and other molecules known to be associated with lymphangiogenesis, we examined the expression of CEACAM-1, LYVE-1, podoplanin, PROX-1, VEGF-C, and VEGFR-3 by qRT-PCR (**Figure 4**). As can be seen, MfAg did not alter expression of any of these molecules, suggesting that microfilariae-induced LEC differentiation is not related to expression of these particular markers.

### Gene expression analysis identifies cell-cell interaction pathways in MfAg-induced tube formation

**Figure 5:**
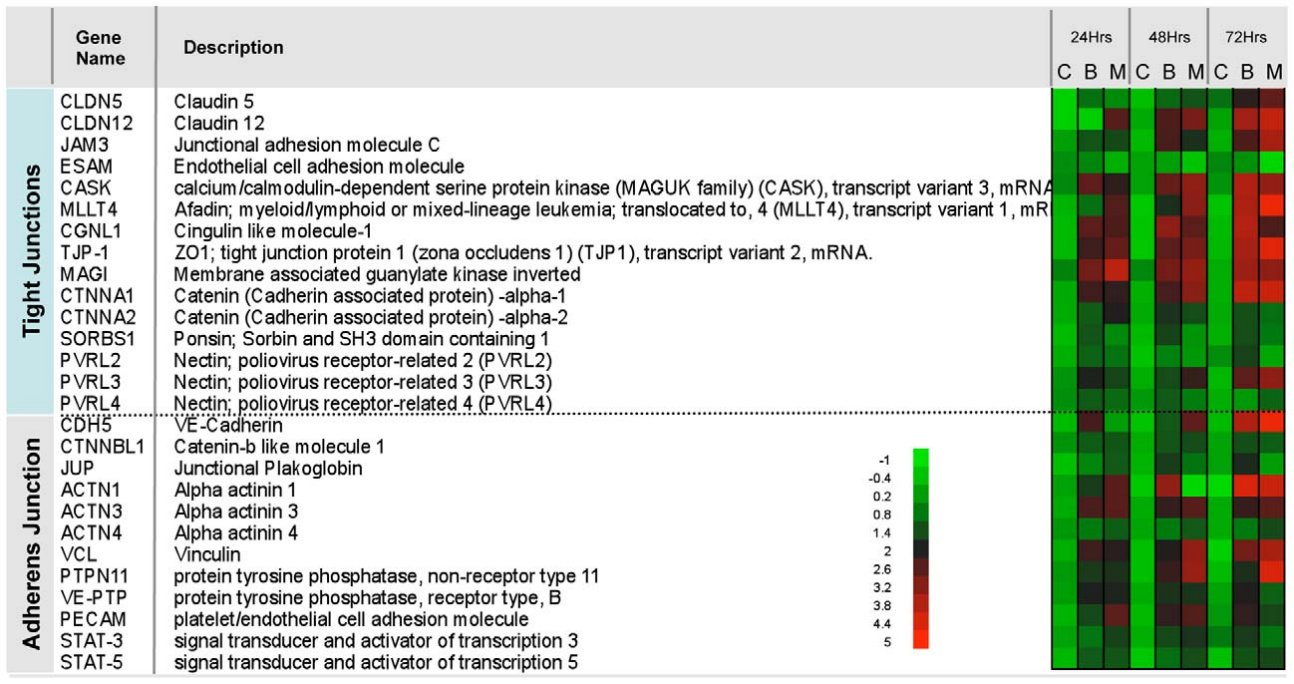
Expression of junction molecules by LEC by microarray analysis. Shown in the heat map are the relative expression levels of the genes listed in response to BmA (B) or MfAg (M) at 24-, 48-, and 72-h time points. The control values (C) are the relative expression levels of pooled controls at the time-points listed. The heat map scale is shown in the inset.

To assess more globally the molecules involved in LEC differentiation induced by filarial pathogens, we carried out microarray studies comparing unstimulated LEC and MfAg-stimulated LEC. Notably in these analyses (**Tables 1** **and** **2**), expression of a number of genes associated with lymphangiogenesis (e.g. CADM1, TBK-1, GJA4, CD36, ANGPTL4, ICAM-2, MMP-1, CASK, TGFBI, VIP, CTGF, OLFML3, SORBS2, GJA1, MARCKS) were found to be altered by MfAg compared with the unstimulated control; many of these (based on pathway analysis) were associated with network functions of cellular development, cellular assembly and organization, hematologic system development and function, and cancer/tumor morphology. Furthermore, the results from these microarrays indicated that the expression of the molecules involved in maintenance of endothelial cell-cell contact (tight junctions and adherence junctions) are altered upon stimulation with filarial antigens (**Figure 5**). In addition, when qRT-PCR was performed independently on the majority of the genes shown to have filarial-induced alterations in gene expression, there was a strong relationship between the expression levels assessed by microarray and those assessed independently by qRT-PCR (**Figure S2**).

**Table 1:** List of MfAg induced 2-fold upregulated genes.

Name	Description	Fold Change
CADM1	cell adhesion molecule 1	12.295
TBK1	TANK-binding kinase 1	8.664
RBP7	retinol binding protein 7, cellular	7.346
GJA4	gap junction protein, alpha 4, 37kDa	7.341
ADFP	adipose differentiation-related protein	7.321
CYP1B1	cytochrome P450, family 1, subfamily B, polypeptide 1	6.788
PLTP	phospholipid transfer protein	6.727
CASK	calcium/calmodulin-dependent serine protein kinase (MAGUK family)	6.476
ANGPTL4	angiopoietin-like 4	6.422
CD36	CD36 molecule (thrombospondin receptor)	6.16
TGFBI	transforming growth factor, beta-induced, 68kDa	5.93
ENC1	ectodermal-neural cortex (with BTB-like domain)	5.844
ACSL1	acyl-CoA synthetase long-chain family member 1	5.641
C19ORF2	chromosome 19 open reading frame 2	5.468
GUSB	glucuronidase, beta	4.897
IQGAP2	IQ motif containing GTPase activating protein 2	4.857
FAM14A	family with sequence similarity 14, member A	4.646
GUCY1A3	guanylate cyclase 1, soluble, alpha 3	4.643
MMP1	matrix metallopeptidase 1 (interstitial collagenase)	4.624
HDAC5	histone deacetylase 5	4.563
KIF21A	kinesin family member 21A	4.547
RPESP	RPE-spondin	4.525
SYT9	synaptotagmin IX	4.359
IL1R1	interleukin 1 receptor, type I	4.332
PDK4	pyruvate dehydrogenase kinase, isozyme 4	4.305
NP	nucleoside phosphorylase	4.305
NNMT	nicotinamide N-methyltransferase	4.263
GADD45G	growth arrest and DNA-damage-inducible, gamma	4.26
FAM46A	family with sequence similarity 46, member A	4.249
CREB5	cAMP responsive element binding protein 5	4.216
UCHL1	ubiquitin carboxyl-terminal esterase L1 (ubiquitin thiolesterase)	4.158
CH25H	cholesterol 25-hydroxylase	4.05
6-Sep	septin 6	4.045
VIP	vasoactive intestinal peptide	4.008
ATP5G2	ATP synthase, H+ transporting, mitochondrial F0 complex, subunit C2 (subunit 9)	3.994
CECR1	cat eye syndrome chromosome region, candidate 1	3.992
CIB1	calcium and integrin binding 1 (calmyrin)	3.853
ICAM2	intercellular adhesion molecule 2	3.818
RPS9	ribosomal protein S9	3.816
AKR1C2	aldo-keto reductase family 1, member C2 (dihydrodiol dehydrogenase 2; bile acid binding protein; 3-alpha hydroxysteroid dehydrogenase, type III)	3.813
BCL11A	B-cell CLL/lymphoma 11A (zinc finger protein)	3.766
PLA2R1	phospholipase A2 receptor 1, 180kDa	3.735
CLDN23	claudin 23	3.727
PRELID1	PRELI domain containing 1	3.678
FECH	ferrochelatase (protoporphyria)	3.671
OAS1	2′,5′-oligoadenylate synthetase 1, 40/46kDa	3.671
MYL6B	myosin, light chain 6B, alkali, smooth muscle and non-muscle	3.638
GDF15	growth differentiation factor 15	3.638
FLOT1	flotillin 1	3.63
ACADVL	acyl-Coenzyme A dehydrogenase, very long chain	3.613
ZNF197	zinc finger protein 197	3.573
RPL18A	ribosomal protein L18a	3.497
KIAA1271	virus-induced signaling adapter	3.492
ATP8A1	ATPase, aminophospholipid transporter (APLT), class I, type 8A, member 1	3.482
TSKU	tsukushin	3.463
HMOX1	heme oxygenase (decycling) 1	3.449
ID4	inhibitor of DNA binding 4, dominant negative helix-loop-helix protein	3.378
ALDH2	aldehyde dehydrogenase 2 family (mitochondrial)	3.359
MCTS1(includesEG:28985)	malignant T cell amplified sequence 1	3.338
HERC5	hect domain and RLD 5	3.26
SPTBN1	spectrin, beta, non-erythrocytic 1	3.211
CHST3	carbohydrate (chondroitin 6) sulfotransferase 3	3.134
INHBB	inhibin, beta B	3.125
DYRK2	dual-specificity tyrosine-(Y)-phosphorylation regulated kinase 2	3.108
PAG1	phosphoprotein associated with glycosphingolipid microdomains 1	2.988
MGC11324	lung cancer metastasis-associated protein	2.961
MGMT	O-6-methylguanine-DNA methyltransferase	2.946
RHOU	ras homolog gene family, member U	2.944
MGLL	monoglyceride lipase	2.944
DCLRE1C	DNA cross-link repair 1C (PSO2 homolog, S. cerevisiae)	2.912
NOC2L	nucleolar complex associated 2 homolog (S. cerevisiae)	2.902

**Table 2:** List of MfAg induced 2-fold down-regulated genes.

Name	Description	Fold Change
CTGF	connective tissue growth factor	8.586
ACTB	actin, beta	8.129
OLFML3	olfactomedin-like 3	6.238
SORBS2	sorbin and SH3 domain containing 2	5.134
PEG10	paternally expressed 10	4.721
IER3	immediate early response 3	4.287
RRM2	ribonucleotide reductase M2 polypeptide	4.095
GJA1	gap junction protein, alpha 1, 43kDa	4.093
RPL24	ribosomal protein L24	4.056
MARCKS	myristoylated alanine-rich protein kinase C substrate	3.983
SNCAIP (includes EG:9627)	synuclein, alpha interacting protein (synphilin)	3.784
GFRA1	GDNF family receptor alpha 1	3.663
SC4MOL	sterol-C4-methyl oxidase-like	3.338
DHCR24	24-dehydrocholesterol reductase	3.31
DDAH1	dimethylarginine dimethylaminohydrolase 1	3.297
ERBB4	v-erb-a erythroblastic leukemia viral oncogene homolog 4 (avian)	3.285
SEMA3C	sema domain, immunoglobulin domain (Ig), short basic domain, secreted, (semaphorin) 3C	3.236
GCNT1	glucosaminyl (N-acetyl) transferase 1, core 2 (beta-1,6-N-acetylglucosaminyltransferase)	3.224
USP21	ubiquitin specific peptidase 21	3.123
NEBL	nebulette	3.055
PLA2G4B	phospholipase A2, group IVB (cytosolic)	2.979
EPS15L1	epidermal growth factor receptor pathway substrate 15-like 1	2.86
NAPE-PLD	N-acyl-phosphatidylethanolamine-hydrolyzing phospholipase D	2.678
CASP4	caspase 4, apoptosis-related cysteine peptidase	2.519

### Microfilariae inhibit tissue inhibitors of matrix metalloproteinase (TIMP)-1 and TIMP-2 expression and increase matrix metalloprotease (MMP) activity

**Figure 6:**
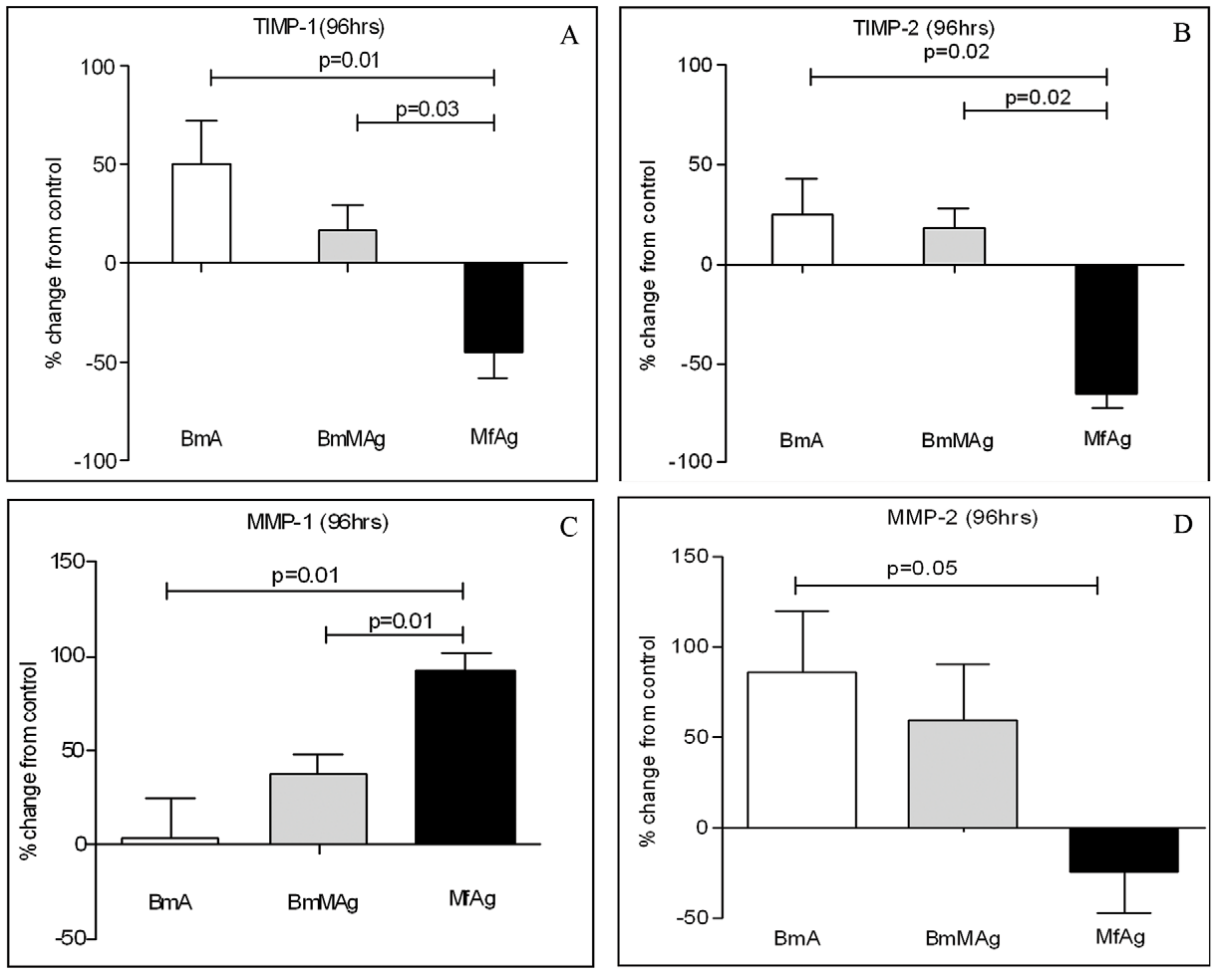
Filarial antigens induce metalloproteases MMP-1 and MMP-2. Shown are the levels of TIMP-1 (Panel A) and TIMP-2 (Panel B), MMP-1 (Panel C) and MMP-2 (Panel D) upon stimulation with various filarial antigens. Data are expressed as the mean (+95% CI) % change from control (media) for 3 independent experiments.

With the microfilaria-induced increases in gene expression associated with pathways involved in cell-cell contact, tissue remodeling, and angiogenesis, we next assessed the levels of the proteins (gene products) associated with these processes. Following stimulation of LEC with MfAg, it could be shown clearly that TIMP-1 and TIMP-2 levels were significantly inhibited (up to 75%) (**Figure 6A and 6B**) compared with either BmA (TIMP-1: *P* = 0.01; TIMP-2: *P* = 0.02) or BmMAg (TIMP-1: *P* = 0.03; TIMP-2: *P* = 0.02) -exposed LEC. There was no significant up- or downregulation of angiopoietin-2 (Ang-2), fibroblast growth factor (FGF), platelet-derived growth factor (PDGF), vascular endothelial growth factors - VEGF-A, VEGF-C, or VEGF-D by the parasite antigens. Other growth factors such as heparin-binding epidermal growth factor (HB-EGF), hepatocyte growth factor (HGF), keratinocyte growth factor (KGF), and thrombopoietin (TPO) were barely (or not) detectable (data not shown).

As MMP are regulated by the TIMPs, we next analyzed the parasite antigen-exposed LEC for the presence of active MMPs (MMP-1, -2, -3, -7, -8, -9, -10, and 13). Of all the MMPs assayed, only MMP-1 and MMP-2 were consistently altered by filarial antigen stimulation, with MfAg preferentially inducing expression of MMP-1. As can be seen, MfAg increased expression of MMP-1 significantly more compared with adult antigens BmA (*p* = 0.001) and BmMAg (*p* = 0.001) (**Figure 6C**). The adult antigens induced higher levels of MMP-2 by the LEC compared with MfAg (*p* = 0.05) (**Figure 6D**). These results suggest that MfAg-induced LEC differentiation may be associated with induction of MMP-1 and inhibition of TIMP-1 and TIMP-2.

### Filarial antigens do not induce permeability of LEC

**Figure 7:**
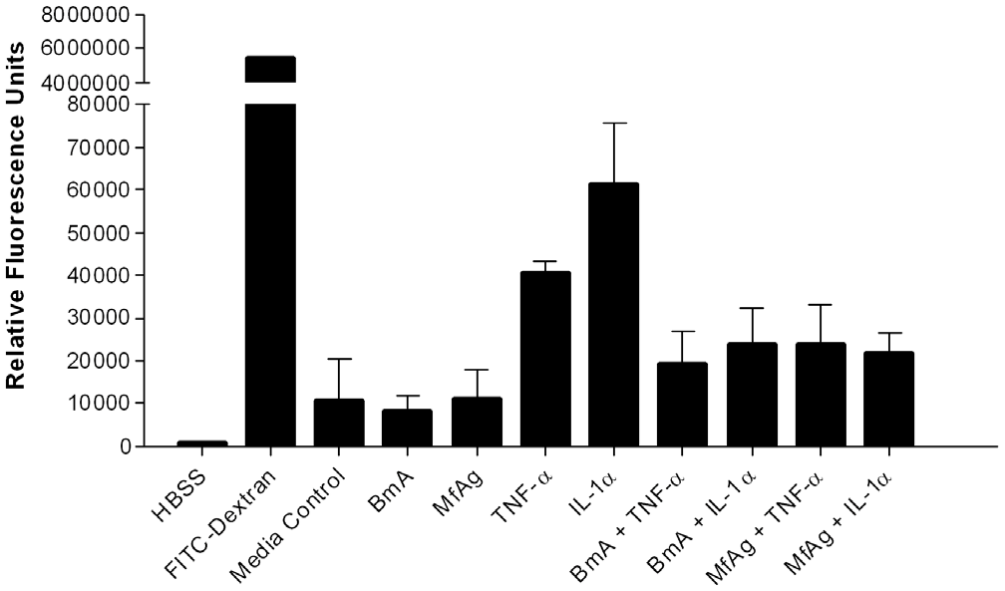
BmA and MfAg inhibit the TNF-α or IL-1α induced hyper-permeability of LEC monolayers. Shown are the mean (+95% CI) relative fluorescence units of 4 independent permeability assays through LEC confluent monolayers.

Lymphedema in filaria-infected individuals is assumed to result from the lymphatic endothelium being rendered hyperpermeable, but the results from the microarrays (**Tables 1** **and** **2** **and** **Figure 5**) suggest an increase in the cell-cell junction genes coding for the proteins that would actually limit trans-endothelial transport. Nevertheless, we evaluated the antigen-induced permeability of LEC *in vitro*. As shown (**Figure 7**), monolayers of LEC exposed to BmA or MfAg for 16–20h had permeability measurements similar to those of unstimulated controls, whereas known inducers of permeability (e.g., TNF-α, IL-1α) were capable of inducing significant trans-endothelial permeability. These data suggest that the changes induced by filarial antigens do not increase trans-endothelial passage of small molecules. In addition, BmA or MfAg were able to impede the permeability effect of TNF-α and IL-1α.

## Discussion

The pathologic hallmark of lymphatic filariasis is lymphedema and elephantiasis that has been associated with lymphatic dilatation, tortuosity, and obstruction. It is known, however, that individuals carrying adult worms (with or without microfilaremia) may have lymphangiectasia, acute lymphangitis, and lymphedema of the extremities [17]. Further, it has been postulated that with sufficient infection chronicity, individuals who harbor adult *Wuchereria bancrofti* will develop lymphangiectasia in the vicinity of the worm nests [20]. Although it is not clear what induces the lymphatic dysfunction in filaria-infected individuals, previous studies have shown that filarial antigens do not induce proliferation of HUVEC *in vitro* and, in fact, inhibited their proliferation [21]. More recent findings, implicate the role of VEGF family members in filarial pathology [25],[26],[27],[28]. The availability of markers (Prox-1, podoplanin, LYVE-1, VEGFR-3, among others) to differentiate LEC from blood vascular endothelial cells has facilitated the study of LEC directly. Although LEC have a lower turnover rate compared with blood vascular endothelial cells, on appropriate stimulation *in vivo*, LEC are capable of proliferating and migrating to organize into new lymphatic vessels (lymphangiogenesis) [29]. That this process occurs in response to filarial infection/antigens is demonstrated by our findings that filarial antigens specifically induce LEC proliferation and/or differentiation and do so in an antigen-specific manner. Compared to the LECs, under identical serum deprived conditions, VEGF-A induced proliferation of the HUVEC was similar to that of filarial antigens. It is not clear if the observed lower proliferative response of the HUVECs (compared to the LEC) to both the filarial antigens and VEGF-A reflects on their maximum proliferative capacity *in vitro*. Further, the failure of the filarial antigens to stimulate both the LECs and HUVECs in the presence of serum supplemented media compared to unstimulated controls probably reflects on various growth factors in the sera masking the proliferative capacity of the antigens, which could possibly account for similar observation with HUVECs previously [21]. Though VEGF-C (native or the mutant form of VEGFC-156Ser) specifically activates LEC, it was unable to induce significant proliferation of the LEC at the concentration similar to VEGF-A (25ng/ml). Our data show that, unlike the schistosome antigens (SEA or SWAP) that have no effect on LEC (present study) but do induce the proliferation of HUVECS (**Figure 1C, 1D**) [22], both adult filarial antigens and serum from patients with lymphatic filariasis induce LEC proliferation. As has been shown by others [25],[26],[27],[28], it is possible that circulating levels of pro-lymphangiogenic factors or circulating parasite antigens (>32,000 Og4C3 units in plasma of infected individuals, data not shown) themselves could be stimulating LECs *in vivo*. In addition, MfAg appears to induce lymphatic vessel differentiation and remodeling that may be aided by the alteration of the extracellular matrix.

The lymphangiogenic response requires both upregulation of lymphangiogenic factor expression and downregulation of the inhibitors of lymphangiogenesis [30] (typically involved in degradation and synthesis of the extracellular matrix components [31],[32]). Although VEGF family members have been considered the prime mediators of lymphangiogenesis, many other mediators have been identified that can also influence the lymphatic vasculature [33],[34],[35],[36],[37]. Among these HGF, bFGF, PDGF, angiopoeitins, and IGF-1 are known inducers and/or regulators of lymphangiogenesis; however, we were unable to detect any significant increase in the levels of HGF, b-FGF, ANG-2 or PDGF in the culture supernatants of LEC stimulated with MfAg. This however does not preclude the release of VEGF in *in vivo* conditions by the immune cells such as the macrophages that could aid in the filarial pathology. Thus, the host factors mediating lymphangiogenesis and lymphatic differentiation awaits clarification.

Typically, confluent monolayers of lymphatic endothelium in the presence of growth factors form tubular networks that are similar in their structural arrangement to lymphatic capillaries *in vivo* [38]. EM analysis of these tubular structures suggested that the flattened EC form a luminal space comprising one to several EC. Typical studies on lymphangiogenesis utilize collagen- or gelatin-coated culture plates or, for 3-dimensional studies, Matrigel™; however, filarial antigen-induced tube formation did not require any pre-existing matrix, suggesting that laminin and collagen type IV (major components of Matrigel™) are not absolutely essential for differentiation of LEC *in vitro* as has been demonstrated in previous *in vitro* studies [39],[40]. This is indicative of the capillary EC inherent capacity to produce and secrete factors that can aid in tissue remodeling (e.g. MMP) [41]. The activity of MMP is further controlled by endogenous TIMP that can bind to zymogens or inhibit the activated forms of MMP [42],[43],[44],[45]. TIMP-1 and TIMP-2 are the best-known inhibitors that can suppress capillary endothelial cell function *in vitro*. The filarial antigen-induced tube formation seen in the present study seemed most dependent on regulation of TIMP-1 and TIMP-2 that were, in turn, associated with increased levels of MMP-1 and MMP-2. Preliminary data suggest that addition of exogenous TIMP-1 and TIMP-2 inhibit the antigen-induced tube formation to a considerable extent (data not shown). It remains to be seen how TIMP and MMP regulate LEC activity upon stimulation with filarial antigens, although their role in development of collateral/accessory lymphatics in cats infected with *Brugia pahangi* [46] and in humans with lymphatic filariasis has been postulated previously.

A characteristic feature of long-term filarial infection in humans and animals is the fibrosis and cellular hyperplasia in and around the lymphatic walls. Infection with the parasites for long periods results in the fibrosis of the infected lymph nodes, which eventually become non-functional and are bypassed by new lymphatic vessels [46]. Several animal studies [47],[48],[49] have previously shown that EC from infected animals had a decreased number of vesicles (which presumably transport fluid) and an increase in the number of vacuoles (which presumably results from cell damage), suggesting that EC lining the lymphatics inhabited by the filarial worms are affected by the parasites. The injury probably renders these EC less effective in transporting edematous fluid and thereby contributes to the edema and collagen accumulation. Studies on LEC permeability (as an indirect measure of their transport capability) demonstrated that, upon stimulation with filarial antigens, LEC are not rendered hyperpermeable but rather are made less leaky, in large part by the induction of adhesion molecules involved in maintaining cell junctions. A recent study using *Dirofilaria immitis* antigen on EC also suggests that antigen-treated monolayers do not differ from the unstimulated controls in the trans-endothelial passage of FITC-Dextran [50]. These data suggest that the lymphedema associated with filarial infection is associated with an inability of the parasite-exposed lymphatics to resorb interstitial fluid rather than for fluid to pass through the LEC junctions abnormally. Alternatively, the proinflammatory responses (IL-1, TNF-α) seen in immune effector cells from patients with chronic lymphatic obstruction [51] could themselves render the LEC hyperpermeable. The proliferative effects of the adult antigens and more pronounced tube-formation activity of the microfilarial antigens, perhaps is an indication of the lymphatic dilatation/damage seen in the sub-clinical infections. It is not absolutely clear, but the consensus on the progression and development of lymphedema/elephantiasis lies in the death of the parasites that triggers a massive inflammatory response against the parasite products and its endosymbiont wolbachia. By whatever mechanism, the interstitial fluid remains and leads to fibrosis and tissue injury.

Thus, our studies reveal that filarial parasites are capable of inducing lymphangiogenesis *in vitro*, a process that is LEC specific and relates to the excretory-secretory components of the filarial parasites and/or the elevated levels of circulating lymphangiogenic factors [26]. The new vessels formed appear to be associated with intrinsic extracellular matrix modeling and are not related to expression of prototypical markers of lymphangiogenesis seen in tumor biology. Our data also suggest that filarial lymphedema may not be due to parasite-induced alterations in trans-endothelial transport (as has been hypothesized) but may involve host-expressed inflammatory components. With the elucidation of the stage-specific excretory-secretory components of *B. malayi* [52],[53], further studies on the effects of these ES products (and their purified components) on the LECs would enable a clearer understanding of the filarial induced lymphatic dysfunction associated with lymphatic filariasis.

## Materials and Methods

### Cell cultures

Human dermal microvascular endothelial cells (HDMEC), human lymphatic microvascular endothelial cells (here called LEC), and human umbilical vein endothelial cells (HUVEC) were obtained from Cambrex. LEC and HUVEC (together referred to as EC) were cultured in EGM-2MV (Cambrex), serially subcultured, and maintained through 7–8 passages in T75 tissue culture flasks (Sarstedt; Fisher Scientific). All experiments were performed using cells from passages 3–5. The purity of LEC was verified by their cell surface expression of podoplanin, VEGFR-3, and LYVE-1 by flow cytometry and confocal microscopy.

### Measurement of LEC marker expression by flow cytometry, confocal microscopy and quantitative reverse transcriptase real-time PCR (qRT-PCR)

For flow cytometry analyses, the cells were detached from the culture flasks by trypsinization per manufacturer's instructions (Reagent Pack, subculture reagents; Cambrex). The detached cells (for flow cytometry) or adherent cells in 8-well chambered glass slides (Lab-Tek; NUNC) (for confocal microscopy) were stained individually or in combination with mouse monoclonal anti-human LYVE-1 (MAB2089; R&D Systems), anti-human ICAM-1-FITC (BBA20; R&D Systems), anti-human VEGFR3-APC (FAB3492A; R&D Systems), mouse anti-human CD44-PE (BD Pharmingen), or mouse polyclonal anti-human podoplanin (Cell Sciences). Anti-mouse IgG coupled with Alexa Fluor 594 or Alexa Fluor 488 were from BD Pharmingen. Staining with isotype control Ab were performed in parallel. Flow cytometry data were analyzed by FlowJo (Treestar Inc.). Confocal microscopy was carried out using a Leica-SP2-AOBS unit at the NIAID Biologic Imaging Facility.

QRT-PCR was performed on total RNA samples using commercially available assays (TaqMan™; Applied Biosystems) for podoplanin, LYVE-1, Prox-1, VEGF-C, VEGFR-3, MRC-2, CEACAM, and CD44 on an ABI Prism 7900 sequence detection system (Applied Biosystems). All data are initially expressed as fold change from the endogenous control (18s rRNA). Details of the genes targeted for assessment of quantitative RT-PCR are listed in **Table S1**.

### Antigens, adult parasites, and microfilariae

Soluble extracts of *B. malayi* adult parasites (BmA), adult male (BmMAg), and microfilariae (MfAg) were prepared as described previously [54], as were soluble extracts of *Schistosoma mansoni* (SWAP) and egg antigen (SEA) [22]. *B. malayi* antigens were used both because of their ability to be maintained in animal hosts and because of their extensive homology to the other lymphatic dwelling filariae, *W.bancrofti* that has as its only host, humans.

Live adult parasites and microfilariae were obtained from the NIH/NIAID-Filariasis Research Reagent Repository Center at the University of Georgia, Athens. The animal procedures were conducted in accordance with ACUC guidelines with the approval from the Institutional Review Boards at the National Institutes of Health and at the University of Georgia. The adult worms were washed thoroughly in RPMI-1640 supplemented with penicillin/streptomycin, 2 mM L-glutamine, and 10 g/l of d-glucose (MF-media). Microfilariae were washed and resuspended in MF-media, layered onto LSM (MP Biomedicals), and centrifuged at 1750 rpm for 20 min. The pellet of live microfilariae was cleared of all contaminating cells by incubation with lysis buffer for 10 min, washed in MF-media, and enumerated.

### Proliferation of LEC and HUVEC

Primary EC were detached from culture flasks by trypsinization and seeded at a density of 5×10^3^ cells/well in 96-well flat-bottomed plates in EGM-2MV media (Cambrex). The cells were starved overnight in endothelial basal media (EBM-2) before being stimulated with various concentrations of BmA, BmMAg, or MfAg. SWAP and SEA were used as nonspecific parasite stimulus. VEGF-A and VEGF-C were used as specific stimulators of EC. Untreated cells with media alone served as negative controls. EC were cultured at 37°C for 96 h in a 5% CO_2_ incubator in EBM-2 media with/without any growth factors. The cells were pulsed with 1 µCi of H^3^-thymidine 16 h prior to harvesting. The cells were harvested at 96 h and the amount of incorporated H^3^-thymidine measured by Wallac scintillation counter (Perkin Elmer).

Proliferation of LEC in the presence of 0.5% to 1% previously well characterized serum from endemic normals (EN), asymptomatic microfilaraemic (MF), and chronic pathology (CP), or control AB was assayed by incorporation of BrDU (Invitrogen) as per the manufacturer's instructions.

### *In vitro* tube formation assays

LEC were seeded at a density of 0.5×10^6^ cells in 6-well plates (Costar; Fisher Scientific) in EGM-2MV media. The cells were starved overnight in EBM-2 media and were stimulated with antigen preparations (BmA, BmMAg, and MfAg) or live parasites. The live adult parasites (3–4 adults/well) or live microfilariae (50,000) were co-cultured with LEC directly or in transwells. Tube formation was observed and imaged using a Zeiss confocal microscope under a PlastDIC lens every 24 h.

### Angiogenic factors

The culture supernatants from LEC stimulated with filarial antigens or live parasites after 24, 48, 72, and 96 h were screened for Ang-2, FGF-b, HB-EGF, HGF, KGF, PDGF-BB, TIMP-1, TIMP-2, VEGF, VEGF-C, VEGF-D, TPO, or MMP-1, -2, -3, -7, -8, -9, -10, and -13 by multiplex arrays (Searchlight). The intra- and inter-assay coefficients of variances for the multiplexed array were observed to be less than 10% and 25% respectively.

### Permeability assays

Permeability assays on LEC were carried out as the manufacturer's instructions using the *in vitro* vascular permeability assay kit (Chemicon Intl.). Briefly, LEC were seeded at a density of 2×10^5^ cells on collagen-coated transwell inserts in a 24-well tissue culture plate. The cells were cultured for 48 h in EGM-2MV medium for confluent monolayers. The cells in the monolayers were starved overnight with EBM-2 basal medium, the media was replaced, and cells were stimulated with antigens for 16–20 h. Permeability was measured based on the amount of FITC-dextran that diffused from the transwell into the lower compartment.

### Microarray analysis

Equal quantities of total RNA from LEC and LEC exposed to MfAg or BmA were converted to aminoallyl-labeled cDNA using the Ovation aminoallyl amplification and labeling system per the manufacturer's instructions (NuGEN Technologies, Inc.). The aminoallyl-labeled cDNA were labeled using Amersham CyDye (Cy3, unstimulated controls; Cy5, stimulated) reactive post-dye protocol (GE Healthcare) and hybridized onto custom microarrays manufactured by the Microarray Research Facility of the NIAID. The human microarray (Hsbb) probe set is based on 70mer oligonucleotides from Qiagen's Human Genome Oligo Set V2.0. A comprehensive gene list is available at http://madb.niaid.nih.gov/cgi-bin/gipo.

Images of the scanned arrays were analyzed, and data were filtered using the criteria of fold-change of ≥2 (upregulation) or ≤2 (downregulation) of the genes compared with unstimulated controls. For categorizing and pathway analysis, ingenuity pathway analysis (IPA, www.ingenuity.com) was used. Selected genes involved in cell-cell junctions were analyzed by qRT-PCR. The details of the primer/probe sets used are listed in **Table S1**.

The microarray data are available at the NCBI GEOS website http://www.ncbi.nlm.nih.gov/geo/query/acc.cgi?acc=GSE15454.

### Statistical analysis

Data analyses were performed using GraphPad PRISM (GraphPad Software, Inc., San Diego, CA). Unless otherwise noted, geometric means (GM) were used for measurements of central tendency. Statistically significant differences between two groups were analyzed using the non-parametric Mann-Whitney U test. Correlations were calculated by Spearman Rank correlation test.

## Supporting Information

Figure S1 Characterization of LEC. LEC were analyzed for expression of lymphatic specific markers (A) podoplanin, (B) VEGFR3, (C) LYVE 1 and for absence of BEC markers (D) CD44 and (E) ICAM. LEC were >98% podoplanin positive and negative for CD44 and ICAM (F and G). The isotype controls are solid peaks (A E). (H-K) Confocal images of LEC showing podoplanin (Alexa Fluor 488) (H), LYVE 1 (Alexa Fluor 594) (I), co localization of podoplanin and LYVE 1 (J), and DIC image of the LEC (K). (L) QRT-PCR expression of LEC specific markers as fold change compared with HDMEC.
(0.56 MB PDF)

Figure S2 Quantitative reverse transcriptase real-time PCR data correlates strongly with expression levels assessed by microarray. Shown is the relationship of individual gene expression (fold change over media) as assessed by microarray (y-axis) and qRT-PCR in response to BmA at 24hrs (A), MfAg at 24hrs (B) BmA at 72 hours (C) and MfAg at 72 hours (D).
(0.05 MB PDF)

Table S1 List of genes targeted for qRT-PCR used to evaluate gene expression.
(0.05 MB DOC)
