# Supplementary figures and images for: Lymphangiogenesis and Lymphatic Remodeling Induced by Filarial Parasites: Implications for Pathogenesis
Source: PLoS Pathog. 2009 Dec 11;5(12):e1000688. doi: 10.1371/journal.ppat.1000688 (PMC2781552; doi:10.1371/journal.ppat.1000688)

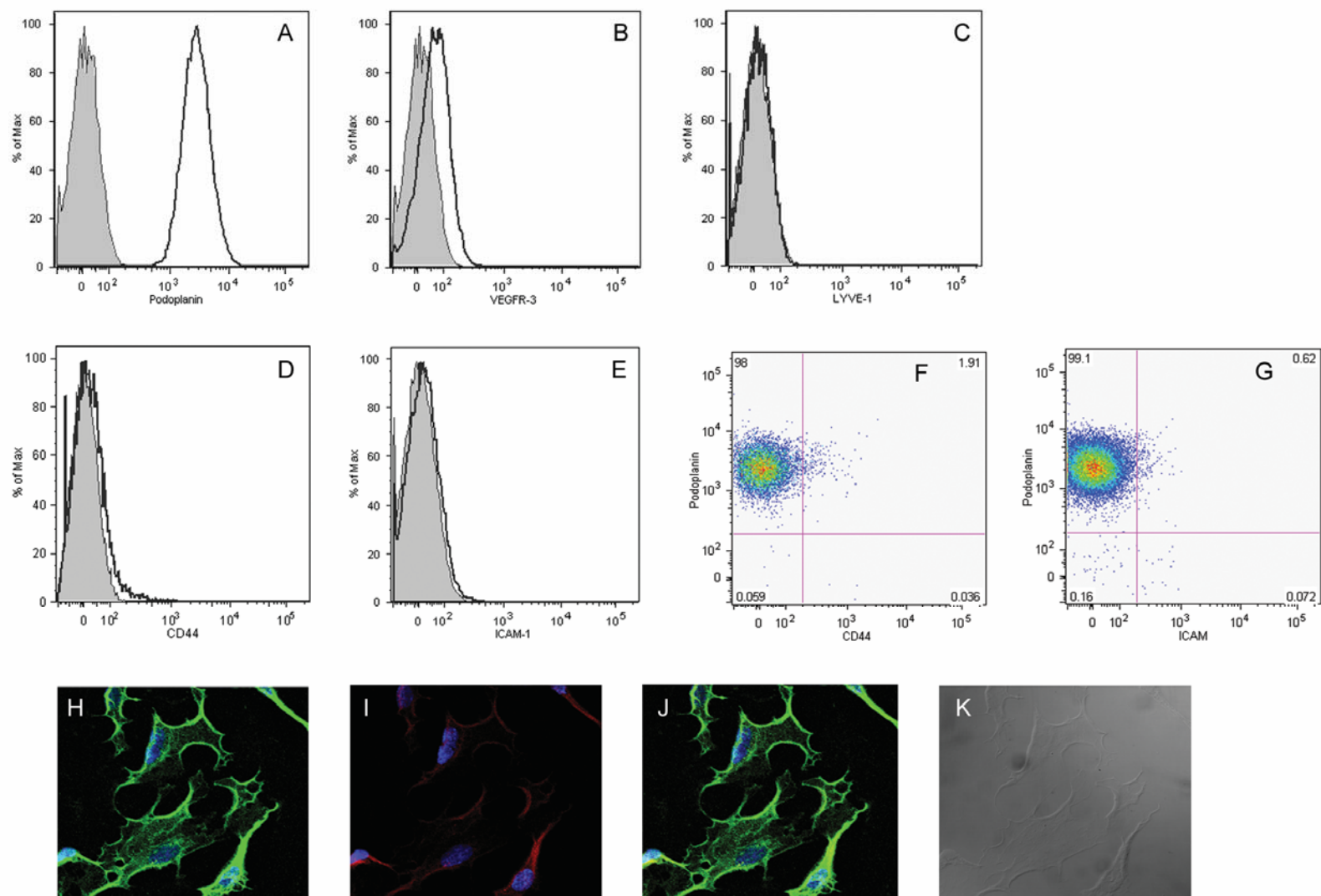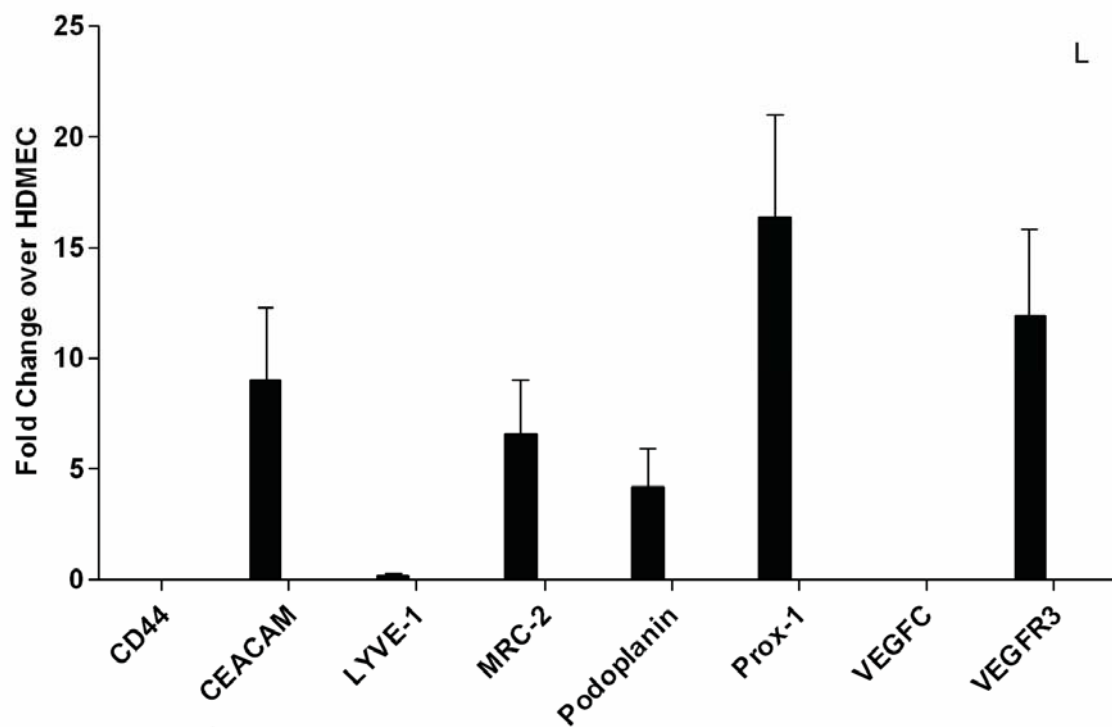

Figure - S1

Supplement: Figure S1 — Characterization of LEC. LEC were analyzed for expression of lymphatic specific markers (A) podoplanin, (B) VEGFR3, (C) LYVE 1 and for absence of BEC markers (D) CD44 and (E) ICAM. LEC were >98% podoplanin positive and negative for CD44 and ICAM (F and G). The isotype controls are solid peaks (A E). (H-K) Confocal images of LEC showing podoplanin (Alexa Fluor 488) (H), LYVE 1 (Alexa Fluor 594) (I), co localization of podoplanin and LYVE 1 (J), and DIC image of the LEC (K). (L) QRT-PCR expression of LEC specific markers as fold change compared with HDMEC. (0.56 MB PDF) [file ppat.1000688.s001.pdf]

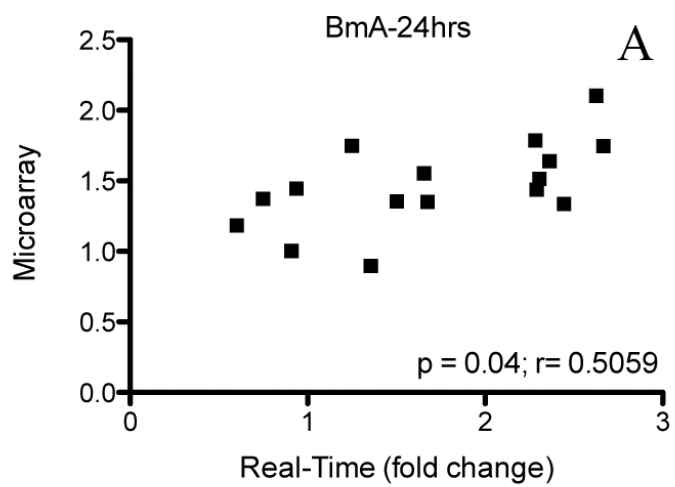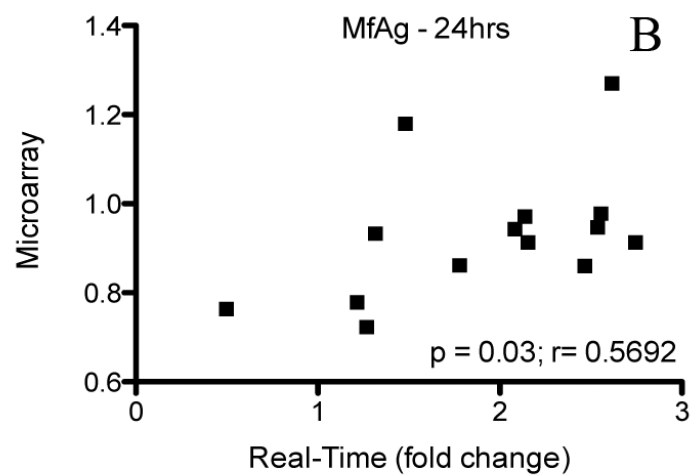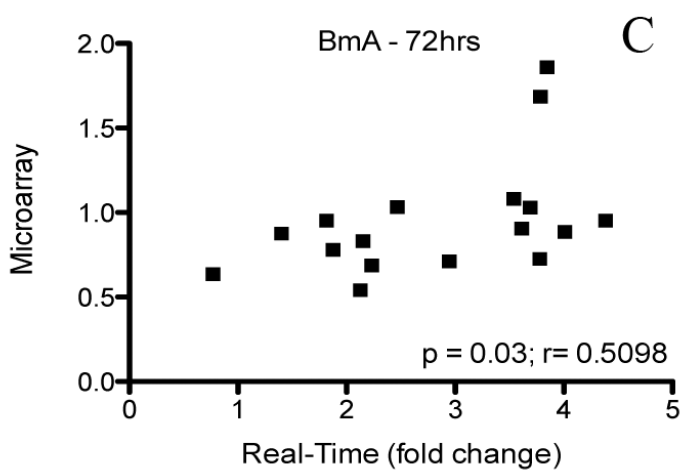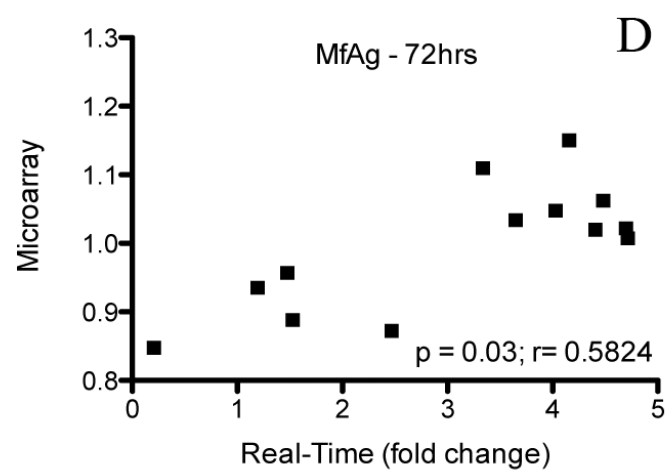

Supplement: Figure S2 — Quantitative reverse transcriptase real-time PCR data correlates strongly with expression levels assessed by microarray. Shown is the relationship of individual gene expression (fold change over media) as assessed by microarray (y-axis) and qRT-PCR in response to BmA at 24hrs (A), MfAg at 24hrs (B) BmA at 72 hours (C) and MfAg at 72 hours (D). (0.05 MB PDF) [file ppat.1000688.s002.pdf]
