# Supplementary material for: Lymphangiogenesis and Lymphatic Remodeling Induced by Filarial Parasites: Implications for Pathogenesis
Source: PLoS Pathog. 2009 Dec 11;5(12):e1000688. doi: 10.1371/journal.ppat.1000688 (PMC2781552; doi:10.1371/journal.ppat.1000688)
Supplement: Table S1 — List of genes targeted for qRT-PCR used to evaluate gene expression. (0.05 MB DOC) [file ppat.1000688.s003.doc]

**Supplemental Table: S1**

List of primer/probe sets for qRT-PCR

| **ABI Catalogue Number** | **Gene ID** | **Gene Description** |
| --- | --- | --- |
| Hs00366764_m1 | TIA-2 | Podoplanin |
| Hs00272659_m1 | LYVE-1 | Lymphatic vessel endothelial hyaluronan receptor 1 |
| Hs00160463_m1 | Prox-1 | Prospero homeobox 1 |
| Hs00153458_m1 | VEGF-C | Vascular endothelial growth factors-C |
| Hs00176607_m1 | VEGFR-3 | Vascular endothelial growth factor receptor-3 |
| Hs00195862_m1 | MRC-2 | Mannose receptor, C type 2 |
| Hs00266109_m1 | CEACAM-1 | Carcinoembryonic antigen-related cell adhesion molecule 1 |
| Hs00153310_m1 | CD44 | Cell surface glycoprotein CD44 |
| Hs01065282_m1 | PECAM-1 | Platelet/Endothelial Cell Adhesion Molecule |
| Hs01361351_m1 | CLDN-5 | Claudin-5 |
| Hs01082669_m1 | CLDN-12 | Claudin-12 |
| Hs00230289_m1 | JAM-3 | Junctional Adhesion Molecule-3 |
| Hs00332781_m1 | ESAM | Endothelial Surface Adhesion Molecule |
| Hs00177620_m1 | CASK | Calcium/calmodulin-dependent serine protein kinase |
| Hs00984486_m1 | MLLT4 | Myeloid/lymphoid or mixed-lineage leukemia (trithorax homolog, Drosophila); translocated to, 4 |
| Hs00262671_m1 | CGNL1 | Cingulin-1 |
| Hs00268480_m1 | TJP1 | Tight junction protein 1 |
| Hs00426896_m1 | CTNNA1 | Catenin (cadherin-associated protein), alpha 1 |
| Hs00189285_m1 | CTNNA2 | Catenin (cadherin-associated protein), alpha 2 |
| Hs00174344_m1 | CDH5 | Cadherin 5 |
| Hs00258305_m1 | CTNNBL1 | Catenin, beta like 1 |
| Hs00158408_m1 | JUP | Junctional Plakoglobin |
| Hs00241650_m1 | ACTN1 | Actinin, alpha 1 |
| Hs00153812_m1 | ACTN3 | Actinin, alpha 3 |
| Hs00245168_m1 | ACTN4 | Actinin, alpha 4 |
| Hs00243320_m1 | VCL | Vinculin |
| Hs00275784_m1 | PTPN1 | Protein tyrosine phosphatase, non-receptor type 1 |
